# Human Brain Endothelial CXCR2 is Inflammation-Inducible and Mediates CXCL5- and CXCL8-Triggered Paraendothelial Barrier Breakdown

**DOI:** 10.3390/ijms20030602

**Published:** 2019-01-30

**Authors:** Axel Haarmann, Michael K. Schuhmann, Christine Silwedel, Camelia-Maria Monoranu, Guido Stoll, Mathias Buttmann

**Affiliations:** 1 Department of Neurology, University of Würzburg, 97080 Würzburg, Germany; schuhmann_m@ukw.de (M.K.S.); stoll_g@ukw.de (G.S.); 2 University Children’s Hospital, University of Würzburg, 97080 Würzburg, Germany; silwedel_c@ukw.de; 3 Department of Neuropathology, University of Würzburg, 97080 Würzburg, Germany; camelia-maria.monoranu@uni-wuerzburg.de; 4 Department of Neurology, Caritas Hospital, 97980 Bad Mergentheim, Germany

**Keywords:** blood–brain barrier, multiple sclerosis, human cerebral endothelial cells, CXCR2, CXCL5, CXCL8, interleukin-8, SB332235

## Abstract

Chemokines (C-X-C) motif ligand (CXCL) 5 and 8 are overexpressed in patients with multiple sclerosis, where CXCL5 serum levels were shown to correlate with blood–brain barrier dysfunction as evidenced by gadolinium-enhanced magnetic resonance imaging. Here, we studied the potential role of CXCL5/CXCL8 receptor 2 (CXCR2) as a regulator of paraendothelial brain barrier function, using the well-characterized human cerebral microvascular endothelial cell line hCMEC/D3. Low basal CXCR2 mRNA and protein expression levels in hCMEC/D3 were found to strongly increase under inflammatory conditions. Correspondingly, immunohistochemistry of brain biopsies from two patients with active multiple sclerosis revealed upregulation of endothelial CXCR2 compared to healthy control tissue. Recombinant CXCL5 or CXCL8 rapidly and transiently activated Akt/protein kinase B in hCMEC/D3. This was followed by a redistribution of tight junction-associated protein zonula occludens-1 (ZO-1) and by the formation of actin stress fibers. Functionally, these morphological changes corresponded to a decrease of paracellular barrier function, as measured by a real-time electrical impedance-sensing system. Importantly, preincubation with the selective CXCR2 antagonist SB332235 partially prevented chemokine-induced disturbance of both tight junction morphology and function. We conclude that human brain endothelial CXCR2 may contribute to blood–brain barrier disturbance under inflammatory conditions with increased CXCL5 and CXCL8 expression, where CXCR2 may also represent a novel pharmacological target for blood–brain barrier stabilization.

## 1. Introduction

The blood–brain barrier (BBB) is a complex multicellular interface between blood and central nervous system (CNS) tissue that tightly controls the exchange of soluble and cellular factors. The border between both compartments is formed by highly specialized endothelial cells connected by tight junctions (TJ). These multi-protein complexes seal the interendothelial clefts under healthy conditions but may open for cytokine, antibody, and immune cell extravasation in a variety of pathophysiological conditions in a highly regulated and dynamic manner [1].

Molecular interactions between leukocytes and brain endothelial cells are regulated by adhesion molecules and chemokines, which are small cytokines with chemoattractant properties that can be categorized into four groups according to the configuration of a conserved cysteine motif, namely C, CC, CXC and CX_3_C [2]. The CXC family comprises 15 ligands (CXCL), seven of which (CXCL1-3, CXCL5-8) contain a glutamic acid (E)–leucine (L)–arginine (R) motif shortly before the first cysteine of the CXC motif (so-called ELR^+^ CXC chemokines), enabling their binding to CXC receptor 2 (CXCR2) [3]. The G-protein coupled receptor CXCR2 can be found on neutrophils, T lymphocytes, and basophils but it is also expressed on non-hematopoietic cells including oligodendrocytes and endothelium [4,5,6].

A disturbance of the BBB plays a pivotal role in the pathogenesis of multiple sclerosis (MS), an inflammatory autoimmune disease of the CNS. In both MS and its animal model, experimental autoimmune encephalomyelitis (EAE), autoreactive T cells cross the impaired BBB in a tumor necrosis factor-α (TNFα)- and interferon-γ (IFNγ)-driven immune response. Focal demyelinating MS lesions are mainly composed of macrophages, dendritic cells, activated microglia, and lymphocytes. Yet, there is increasing evidence that polymorphonuclear cells (PMN) and neutrophil-attracting CXC chemokines substantially contribute to MS pathology: In EAE, serum levels of granulocyte colony-stimulating factor (G-CSF) rise after immunization with myelin antigens, and depletion of neutrophils or G-CSF receptor deficiency prior to disease onset result in an ameliorated disease course [7,8,9]. In addition, neutrophils have been related to breakdown of the blood–spinal cord barrier in EAE animals. Simultaneously, the same group succeeded in detecting neutrophils in CNS vasculature associated with blood–CNS barrier leakage in tissue sections of brain and spinal cord of both patients with MS and patients with neuromyelitis optica spectrum disorder (NMOSD), respectively [10]. CXCL5 and CXCL8 serum and cerebrospinal fluid (CSF) levels have both been reported to be elevated in MS. CXCL8 is increased in patients with untreated relapsing–remitting MS but declines during treatment with interferon-β1a [11]. CXCL5 has been found to be elevated in patients with gadolinium-enhancing magnetic resonance imaging (MRI) lesions, reflecting acute disturbance of the BBB, in comparison to MS patients with inactive disease [8]. This link to disease activity, partly even before clinical manifestation, suggests a potential role of ELR^+^ CXC chemokines in the chronological sequence of MS lesion formation. 

Here, we studied the expression of CXCR2 in healthy brain tissue and MS plaques *in situ* and in resting and inflammation-activated cultured human brain microvascular endothelial cells *in vitro*. We furthermore investigated the effects of CXCR2-binding chemokines CXCL5 and CXCL8 on brain endothelial tight junction morphology and barrier function *in vitro*. 

## 2. Results

### 2.1. CXCR2 is Weakly Expressed in Resting Brain Endothelium but Highly Inducible by Inflammatory Stimuli

**Figure 1:**
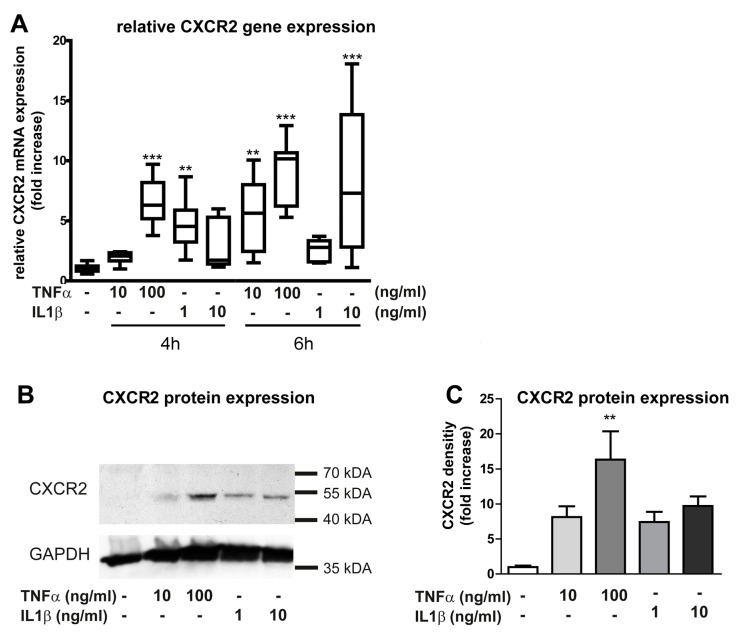
Basal and inflammation-induced CXCR2 expression in human brain endothelium. (**A**) Comparative analysis of CXCR2 mRNA levels by quantitative real-time PCR in hCMEC/D3 after exposure to TNFα or IL1β for 4 or 6 h. Boxplot and whiskers (min to max) of four independent experiments. Statistical analysis by Kruskal–Wallis test followed by Dunn’s post-test. (**B**) Representative example of immunoblotting for CXCR2 protein of hCMEC/D3 whole cell protein extracts after stimulation of cells with TNFα or IL1β for 6 h. Anti-GAPDH (glyceraldehyde-3-phosphate dehydrogenase) served as a loading control. (**C**) Quantification of CXCR2 protein relative to GAPDH in stimulated compared to unstimulated hCMEC/D3 in four independent experiments. Statistical analysis by Kruskal–Wallis test followed by Dunn’s post-test. Asterisks: ** *p* < 0.01; *** *p* < 0.001.

We first analyzed brain endothelial CXCR2 mRNA expression under resting and inflammatory conditions, employing the well-characterized hCMEC/D3 cell line [12]. Quantitative real-time PCR revealed a substantial increase of basal CXCR2 mRNA expression 4 h after starting stimulation with TNFα or interleukin-1β (IL1β; Figure 1A). Dose-dependent induction of CXCR2 mRNA expression by TNFα increased further after 6 h of stimulation, while IL1β showed stronger relative induction at the lower concentration at the earlier timepoint, but stronger induction at the higher tested concentration at the later timepoint in repeated experiments. 

**Figure 2:**
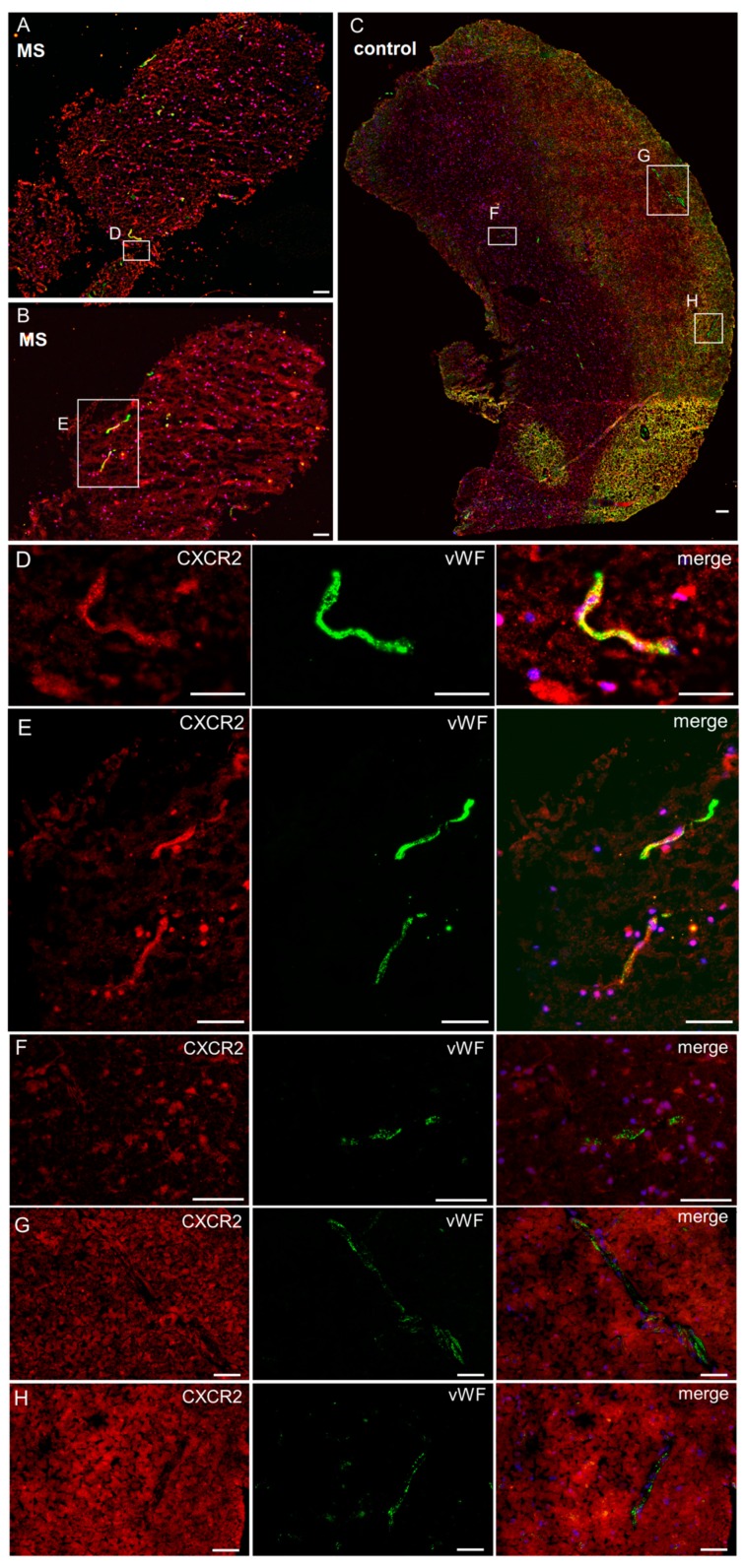
Inflammation-induced upregulation of CXCR2 in human brain tissue. Cryostat sections of stereotactic biopsies from an acute multiple sclerosis (MS) lesion (**A**,**B**,**D**,**E**) or non-inflamed human brain (**C**,**F**–**H**) with immunofluorescence double staining for CXCR2 (red) and the endothelial marker protein von Willebrand factor (vWF) (green). In MS plaques, 116/157 [74%] vessels were CXCR2-positive, while in healthy tissue only 24/104 [24%] vessels were CXCR2-positive (*p* < 0.0001, Fisher’s exact test). Scale bar 50 µm.

At the protein level, CXCR2 expression was barely detectable by Western blotting in resting cells but highly inducible upon stimulation with TNFα or IL1β for 6 h in a concentration-dependent manner (Figure 1B,C). Correspondingly, double immunofluorescence histochemistry for CXCR2 and the endothelial marker protein von Willebrand factor (vWF) of four stereotactic biopsies from healthy (*n* = 2) and inflammatory active MS (*n* = 2) brain tissue revealed an upregulation of CXCR2 protein expression in capillaries of MS plaques (CXCR2-positive vessels in MS: 116/157 [74%] versus control: 24/104 [24%]; *p* < 0.0001, Fisher’s exact test) *in situ* (Figure 2). In summary, these experiments confirmed weak CXCR2 mRNA and protein expression in resting human brain microvascular endothelium and demonstrated their upregulation under inflammatory conditions both *in vitro* and *in situ*.

### 2.2. CXCR2-Binding Chemokines Disturb Paraendothelial Barrier Function in Synergy with TNFα

**Figure 3:**
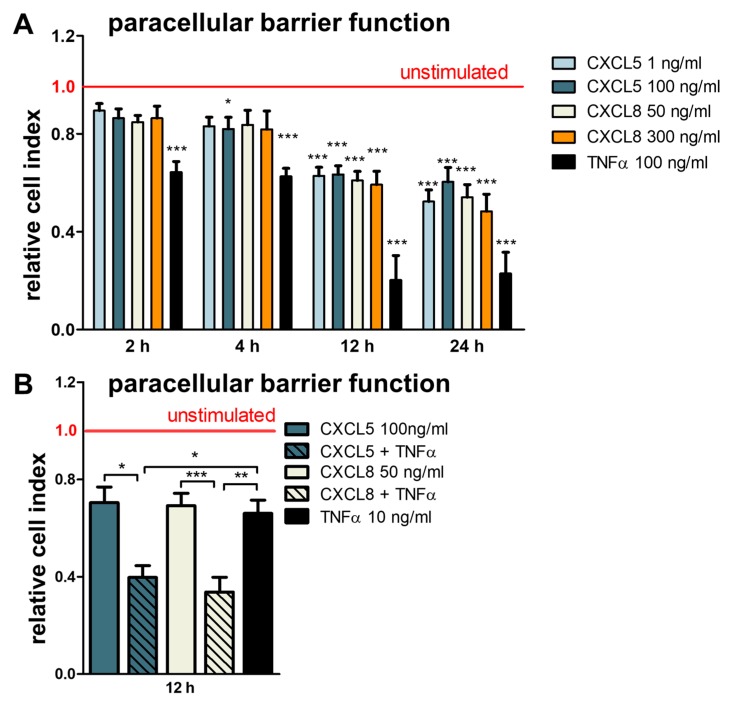
CXCL5 and CXCL8 decrease paracellular barrier function of brain endothelial monolayers in synergy with TNFα. (**A**) Real-time label-free assessment of the Cell Index of hCMEC/D3 using an impedance-based xCELLigence DP system. Stimulation with TNFα (100 ng/mL) served as a positive control. Data represent means and standard errors of the mean (SEM) of six independent experiments run in duplicates, tested by one-way analysis of variance (ANOVA) followed by Bonferroni post-test. (**B**) Effects of CXC chemokines with and without costimulation with TNFα (10 ng/mL) after 12 h. Synopsis of three to four independent experiments run in duplicates. Statistical testing for normal distribution by D′Agostino–Pearson omnibus K2 test, followed by one-way ANOVA and Bonferroni post-test. Asterisks without bars indicate significance compared to unstimulated cells at the respective time points. * *p* < 0.05, ** *p* < 0.01, *** *p* < 0.001.

CXCR2-binding chemokines CXCL5 and CXCL8 were found to be elevated in sera and CSF of patients with inflammatory demyelinating autoimmune disorders of the CNS and were found to increase during relapse, indicating a possible role in the initiation of acute CNS lesions [8,11,13]. Having observed inflammation-inducible CXCR2 expression in brain endothelium, we next monitored paraendothelial barrier function in response to physiologically relevant concentrations of CXCR2-binding chemokines. Using an xCELLigence real-time cell analysis (RTCA) system for tracer-free monitoring of paraendothelial barrier function, both chemokines induced a comparable barrier decrease over 24 h, as—more strongly—did TNFα as a positive control (Figure 3A). In accordance with the observed induction of CXCR2 expression after stimulation with TNFα, the barrier-disturbing effects of CXCL5 and CXCL8 were significantly enhanced by parallel stimulation with 10 ng/mL TNFα (Figure 3B).

### 2.3. CXCL5 and CXCL8 Change Endothelial Monolayer Morphology and Function in a CXCR2-Dependent Manner

**Figure 4:**
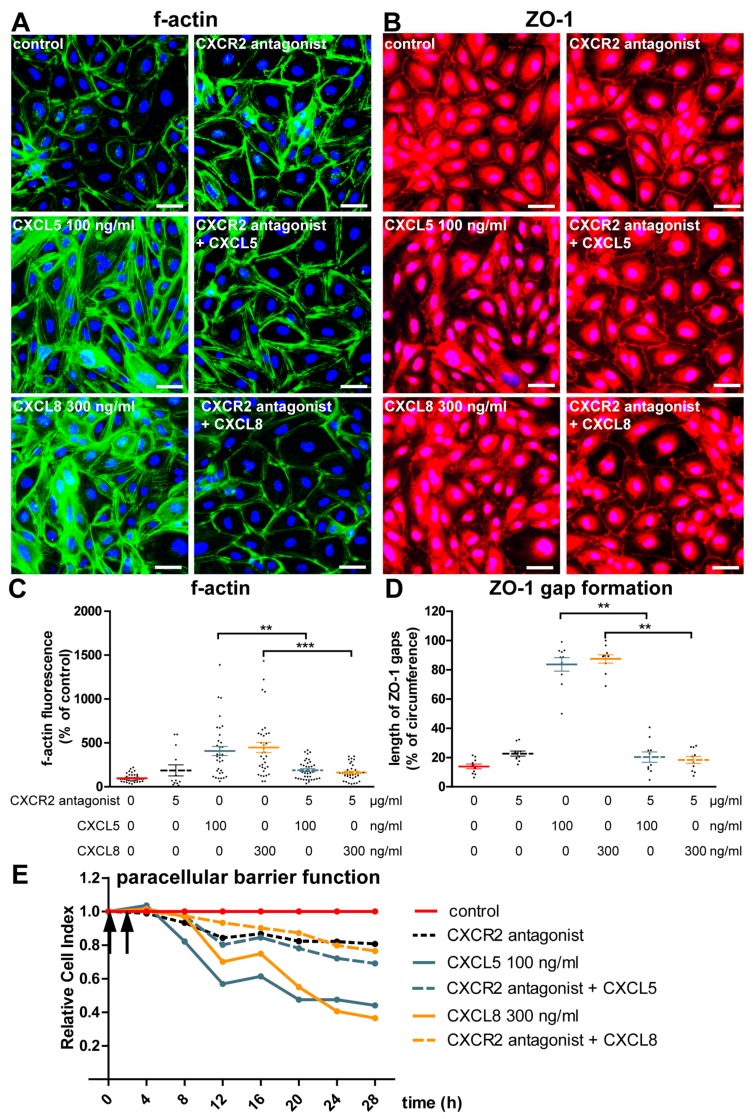
CXCL5 and CXCL8 induce actin stress fibers, downregulate ZO-1, and decrease paraendothelial barrier function via CXCR2. hCMEC/D3 monolayers were pre-incubated with 5 µM CXCR2 antagonist SB332235 or a corresponding concentration of dimethyl sulfoxide (DMSO) for 1 h and subsequently stimulated with CXCL5 or CXCL8 or left untreated for 30 min. (**A**) Visualization of filamentous actin by Alexa Fluor^®^-conjugated phalloidin. (**B**) Immunofluorescent cytochemistry against the tight-junction-associated protein ZO-1. Scale bar = 25 µm. (**C**,**D**) Quantification of relative filamentous actin fluorescence intensity (C) or formation of gaps in ZO-1 staining (D) after stimulation as in (A). Pooled from three to five independent experiments (Kruskal–Wallis test followed by Dunn’s post-test). ** *p* < 0.01; *** *p* < 0.001. For color coding see legend in figure panel (E). (**E**) Real-time monitoring of paracellular permeability after pre-incubation with the CXCR2 antagonist or DMSO control (arrow at the y-axis) for 1 h and subsequent stimulation with CXCL5 and CXCL8 (arrow on the right). Representative of three independent experiments.

The characteristic high transendothelial resistance of brain endothelial monolayers is achieved by the formation of TJ that seal the intercellular clefts. These transmembraneous multi-protein complexes are linked by intracellular adapter proteins such as zonula occludens-1 (ZO-1) to the actin cytoskeleton, which in turn can dynamically regulate TJ morphology and function [14]. Having found disturbance of paraendothelial brain barrier function by CXCR2-binding chemokines, we next studied the effects of CXCL5 and CXCL8 on the morphology of the actin cytoskeleton and of tight junction-associated ZO-1. Within 30 min of chemokine stimulation, a marked redistribution of the subcortical actin network to cytoplasmatic stress fibers could be detected (Figure 4A,C). Simultaneously, circumferential ZO-1 staining was disrupted (Figure 4B,D). Considering that depletion of ZO-1 leads to reduced recruitment of tight junction proteins such as claudin-5 and junctional adhesion molecule-A (JAM-A), this probably reflected destabilization of intercellular junctional complexes [15]. However, additional investigation of the two MS biopsies available for this study, which were characterized by the presence of mononuclear cells and increased vascular CXCR2 expression, did not reveal evidence for albumin extravasation nor for altered expression of ZO-1 or claudin-5 compared to control tissue (not shown). The available MS samples were therefore possibly not suited to corroborate our *in vitro* findings because of the absence of acute blood–brain barrier dysfunction.

While CXCL5 specifically binds to CXCR2, CXCL8 is also capable of activating CXCR1. Furthermore, CXCR2 expression was barely detectable by Western blotting in resting hCMEC/D3 cells in our hands. Thus, to verify that the observed effects were indeed mediated by CXCR2, we subsequently performed experiments with the selective chemical CXCR2 antagonist SB332235 [16]. Pre-incubation with 5 µM SB332235 for 1 h largely prevented chemokine-induced alterations of ZO-1 distribution and actin morphology (Figure 4A–D). Correspondingly, CXCR2 antagonization also partially attenuated the CXCL5- and CXCL8-induced decrease of paraendothelial barrier function (Figure 4E).

### 2.4. CXCL5 and CXCL8 Rapidly Activate Actin-Modulating Kinase Akt/PKB

**Figure 5:**
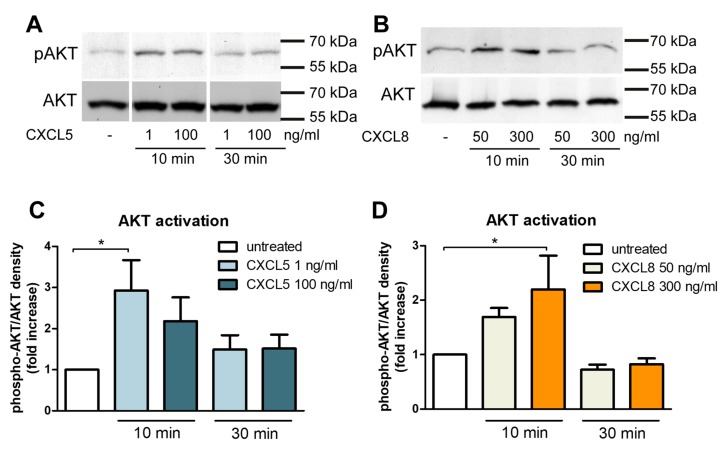
CXCL5 and CXCL8 rapidly and transiently activate Akt/PKB in human brain endothelial cells. Representative Western blot analysis of hCMEC/D3 whole cell protein extracts after stimulation with 1 or 100 ng/mL CXCL5 (**A**) and 50 or 300 ng/mL CXCL8 (**B**) for 10 or 30 min, staining against phosphorylated and total Akt. Quantification of Akt activation upon CXCL5 (**C**) and CXCL8 (**D**) stimulation in four independent experiments. Statistical analysis by one-way ANOVA followed by Bonferroni post-test. * *p* < 0.05.

Activation of CXCR2 by CXC chemokines leads to the dissociation of the heterotrimeric G-proteins and triggers intracellular signaling pathways, including the activation of Akt/protein kinaseB (PKB) [3,17]. Akt/PKB has been reported to be a direct inductor of filamentous actin in hCMEC/D3 by myosin light-chain phosphorylation, thereby regulating endothelial permeability [17]. We therefore studied the activation of Akt/PKB after stimulation with CXCL5 and CXCL8. In accordance with rapid CXCR2-mediated changes of brain endothelial actin morphology and ZO-1 distribution by CXCL5 and CXCL8, we observed rapid transient phosphorylation of Akt/PKB in response to stimulation with CXCL5 and CXCL8 after 10 min, although no significant concentration-dependent effect was seen (Figure 5). 

## 3. Discussion

Here, we demonstrated that human brain endothelial cells show inflammation-inducible expression of CXCR2 both *in vitro* and *in situ* and that this chemokine receptor mediates CXCL5- and CXCL8-induced disturbance of brain endothelial morphology and barrier function *in vitro*. Recent animal data might suggest that this pathway plays a role in MS pathogenesis. Intravital microscopy of spinal cord vessels in mice with myelin oligodendrocyte glycoprotein (MOG_35–55_)-induced EAE revealed that invasion of PMN into the CNS takes place within one day after immunization, precedes clinical disease onset, and is paralleled by extravasation of a low-molecular-weight tracer across the blood-spinal cord barrier [10]. Furthermore, Carlson et al. showed that CXCR2 inhibition prevented BBB breakdown in EAE, despite the presence of activated myelin-specific T cells, potentially arguing for a role of brain endothelial CXCR2 in EAE lesion formation [18]. In contrast to reduced perivascular accumulation of immune cells after CXCR2 inhibition in MOG_35__–__55_-induced EAE, an inhibitory effect was absent when locally applying the endogenous lysophospholipid lysolecithin to chemically induce demyelinating lesions. As shown recently, lysolecithin has direct lipid-disrupting properties [19]. In this case, the local inflammatory response reflects the reaction to, but not the cause of, demyelination and might impair endothelial barrier function, which seems to be a prerequisite for the anti-inflammatory effect of the anti-CXCR2 treatment. In accordance, neutrophil recruitment to the CNS by intraventricular injection of lipopolysaccharide (LPS) was inhibited in chimeric CXCR2 knockout mice reconstituted with the bone marrow of wildtype mice, underlining the potential role of endothelial CXCR2 [20]. However, the same group did not observe a significant reduction of BBB leakage in CXCR2^−/^^−^ mice, thus concluding that the inhibition of CXCR2 in cerebral endothelium does not directly affect vascular permeability. An alternative explanation might be envisaged in our view: LPS is known to rapidly induce apoptosis in endothelial cells, as described in bovine brain endothelial cells *in vitro*, showing positive Annexin V staining after 1 h of incubation [21,22]. Hence, LPS administered directly into the CNS might be such a strong inducer of BBB disruption that effects of CXCR2 inhibition were completely overrun by LPS in the above-mentioned study. Our results in the human system, although only in a cell line *in vitro*, argue for a barrier-disturbing effect of brain endothelial CXCR2.

CXCR2 expression has been reported in various endothelia including human umbilical vein, human dermal microvascular, and human intestinal microvascular endothelial cells [4,23,24,25]. Data concerning CXCR2 expression in human brain endothelium are conflicting: While Berger and colleagues reported primary human brain endothelial cells to be CXCR2-negative, hCMEC/D3 have been demonstrated to show weak expression by both Dywer et al. and Subileau et al., the latter of whom also reported CXCR2 expression in primary human brain endothelial cells [26,27,28]. These heterogeneous results could at least partly be explained by our finding of barely detectable basal CXCR2 expression markedly increased upon inflammatory stimulation, indicating that CXCR2 expression might strongly depend on culture conditions. Direct induction of CXCR2 expression by TNFα or IL1β is plausible also with respect to the fact that CXCR2 is a known nuclear factor kappa-light-chain-enhancer of activated B cells (NFκB) target gene [29]. Nevertheless, Sublieau et al. did not observe TNFα-inducible upregulation of CXCR2 expression in hCMEC/D3, which is in clear contrast to our results with this cell line [28]. In human intestinal microvascular endothelial cells, TNFα- and LPS-triggered CXCR2 upregulation was previously described [24].

In MS lesions, where there is increased TNFα and IL1β expression, the observed CXCR2 upregulation might become particularly relevant, as it may render brain endothelial cells more susceptible to CXC chemokines. Of note, CXCL8 was found to be elevated in the sera of untreated MS patients, and CXCL5 levels correlated with acute BBB dysfunction as indicated by gadolinium-enhanced MRI [8,11]. In addition, CXCL5 was found to be increased in patients with NMOSD, another autoinflammatory disorder of the CNS [13]. In our *in vitro* model of the BBB, CXCL5 and CXCL8 strongly decreased paracellular barrier function via CXCR2 signaling, which is in line with a report of Dywer et al., demonstrating increased transendothelial permeability to high-molecular-weight dextran (40 kDa) after three days of stimulation with CXCL8 [27].

Our *in vitro* observation that a loss of paraendothelial brain barrier properties upon CXCR2 activation was accompanied by endothelial formation of actin stress fibers is consistent with findings in human intestinal and lung microvascular endothelial cells [24,30]. The simultaneous loss of membrane-adjacent ZO-1 which has been shown to result in decreased recruitment of claudin-5 and JAM-A to TJs by others, might reflect a destabilization of intercellular junctional complexes [15]. The two MS brain tissue samples available for this study, which were characterized by an overexpression of CXCR2 compared to control tissue, did not allow corroborating these findings *in situ*, possibly because of the absence of acute BBB dysfunction despite the presence of mononuclear immune cells in our samples. Morphological changes *in vitro* were preceded by rapid transient activation of Akt/PKB in our study, which is known to be a strong modulator of cytoskeletal remodeling and which was found to be activated by CXCR2 in other cell types previously [31]. Interestingly, endothelial cells, including human brain microvascular endothelial cells, are capable of secreting CXCL5 and CXCL8 upon inflammatory stimulation, the latter of which can also be stored in Weibel–Palade bodies and rapidly set free upon endothelial activation [2,28,32,33,34,35]. Together with high brain endothelial expression of glycosaminoglycans, which immobilize secreted chemokines on the cell surface, this might represent an autocrine feedback loop enhancing inflammatory BBB dysfunction [2]. 

Major limitations of our *in vitro* study are the use of immortalized cells, which are less sensitive for changes in paracellular barrier function than primary brain endothelial cells, as well as the use of a monocellular model of the blood–brain barrier lacking astrocytes and pericytes. Yet, we think that the demonstrated increase of endothelial CXCR2 expression in MS plaques, showing that inflammation-induced CXCR2 upregulation is not only an *in vitro* phenomenon but takes place at the neurovascular unit, may underline the pathophysiological relevance of the findings from our model system, although we were not able to corroborate our *in vitro* findings on barrier function with the available MS biopsy material *in situ*.

In summary, we demonstrated that chemokines CXCL5 and CXCL8 disturb paraendothelial brain barrier morphology and function via their inflammation-inducible receptor CXCR2 expressed by human brain endothelium. This might be relevant not only for MS pathogenesis but certainly also for other inflammatory CNS disorders where an overexpression of CXCR2-binding chemokines takes place. Brain endothelial CXCR2 may represent a pharmacological target for BBB stabilization under such pathophysiological circumstances.

## 4. Materials and Methods 

### 4.1. Cell Culture

Immortalized human brain microvascular cells (hCMEC/D3) were purchased from ATCC (Manassas, VA, USA) and cultured at 37 °C/5% CO_2_ in rat collagen-coated flasks with EBM-2 medium (Lonza, Walkersville, MD, USA) supplemented as recommended by the manufacturer. 

### 4.2. Western Blotting 

Whole cell protein extracts were prepared exactly as formerly described [36]. Primary antibodies were used against CXCR2 (1:500, ab14935, Abcam, Cambridge, UK), GAPDH (1:1000; Santa Cruz Biotechnology, Dallas, TX, USA), phospho-Akt (1:1000, #4051, Cell Signaling Technology, Danvers, MA, USA) and Akt (1:1000, #9272, Cell Signaling Technology). Appropriate peroxidase-coupled secondary antibodies were employed with an enhanced chemoluminescence system (Perkin Elmer, Waltham, MA, USA).

### 4.3. xCELLigence Assay 

For label-free real-time assessment of transendothelial resistance, cells were seeded on gold electrode plates in an ACEA xCELLigence DP system (San Diego, CA, USA), which records the impedance changes compared to the background of cell-free electrodes at three different alternating current frequencies expressed as the dimensionless Cell Index (CI), where CI = (impedance at time point n − impedance in the absence of cells)/nominal impedance value [37]. The CI correlates to the transendothelial electrical resistance but does additionally reflect the capacitance of the cell layer. When confluent, as evidenced by a plateau of the CI, the cells were stimulated in duplicates with recombinant human CXCL5, CXCL8, or TNFα (all from PeproTech, Rocky Hill, NJ, USA) or left without stimulation as indicated. For antagonization of CXCR2, the cells were pre-incubated with 5 µM SB332235 (Tocris, Bristol, UK) or a control containing an equivalent concentration of DMSO for 1 h. To compare multiple experiments, the CI of the stimulated cells was related to the CI of the unstimulated control at the indicated time point and shown as the relative CI.

### 4.4. Immunocytochemistry 

The cells were cultured in rat collagen-coated 24-well plates and stimulated as indicated. After rinsing, the cells were fixed and permeabilized with 3.7% paraformaldehyde (PFA) plus 0.1% Triton X-100 for 10 min at room temperature and then blocked with donkey serum (1:100) for 1 h. Thereafter, an anti-ZO-1 antibody (1:100, #617300, Invitrogen, Waltham, MA, USA) and a corresponding secondary anti-rabbit-Cy3 antibody were incubated for 1 h each at room temperature. Filamentous actin was visualized by Alexa Fluor 488-conjugated phalloidin (1:50, A12379, Invitrogen, Waltham, MA, USA). Finally, an anti-fading agent was added. Images were taken with a Leica DMi8 inverted microscope (Wetzlar, Germany) at fixed exposures. For f-actin quantification, fluorescence intensity was quantified in at least four areas per condition of five independent experiments using ImageJ version 1.46r (https://imagej.nih.gov/ij/download/). For quantification of ZO-1, using ImageJ version 1.51k (https://imagej.nih.gov/ij/download/), we divided the length of gaps in the ZO-1 staining by the cell circumferences of at least two view fields per condition of three independent experiments.

### 4.5. Immunohistochemistry 

Cryostat sections of four stereotactic brain biopsies (2× healthy CNS tissue, 2× inflammatory MS lesions) were obtained from the local Department of Neuropathology. The individuals had previously given consent to the use of diagnostic CNS biopsies also for research purposes. Our research was conducted in accordance with local legal regulations, and consent was obtained from the local ethics committee of the Faculty of Medicine at the University of Würzburg (approval number: 99/11, approval date: 13 October 2011). Cryopreserved tissue was fixed with 3.7% PFA, blocked with donkey serum (1:100) for 1 h at room temperature, and subsequently incubated with rabbit anti-CXCR2 antibody (1:250, #ab14935, Abcam, Berlin, Germany) or mouse anti-vWF antibody (1:500, M0616, DAKO (Agilent), Santa Clara, CA, USA) overnight at 4 °C. Subsequently, slices were stained with corresponding Cy2- and Cy3-conjugated secondary antibodies for 1 h. Images were taken with a Leica DMi8 inverted microscope.

### 4.6. Quantitative Real-Time PCR

Total RNA was isolated using Trizol reagent (Invitrogen, Waltham, MA, USA) according to the instructions of the manufacturer. Afterwards, cDNA was synthetized with the TaqMan^®^ Reverse Transcription kit (Applied Biosystems, Carlsbad, CA, USA) and subjected in triplicates to quantitative real-time PCR using TaqMan^®^ reagents and the FAM-MBG inventoried primers Hs01891184_s1 (CXCR2) and Hs02786624_g1 (GAPDH) in a StepOne plus system (all from Applied Biosystems, Carlsbad, CA, USA). Relative changes in gene expression were normalized to GAPDH as an internal control.

### 4.7. Statistics

Statistical analysis was performed with GraphPad PRISM Version 5.04 (La Jolla, CA, USA). For comparison of multiple groups, we performed the non-parametric Kruskal–Wallis test followed by Dunn’s post-test. In case of normal distribution as tested by the D′Agostino–Pearson omnibus K2 test, one-way ANOVA was followed by the Bonferroni post-test. For comparison of CXCR2 positivity between immunohistochemical stained samples, we chose Fisher’s exact test. A *p* value < 0.05 was considered statistically significant. Asterisks indicate levels of significance (* *p* < 0.05; ** *p* < 0.01; *** *p* < 0.001).

## Abbreviations

ANOVA	analysis of variance
BBB	blood–brain barrier
CI	cell index
CNS	central nervous system
CSF	cerebrospinal fluid
CXCL	(C-X-C) motif ligand ligand
CXCR	CXC receptor
EAE	experimental autoimmune encephalomyelitis
GAPDH	glyceraldehyde-3-phosphate dehydrogenase
G-CSF	granulocyte-colony stimulating factor
IFN	interferon
IL	interleukin
JAM-A	junctional adhesion molecule-A
LPS	lipopolysaccharide
MOG	myelin oligodendrocyte glycoprotein
MRI	magnetic resonance imaging
MS	multiple sclerosis
NMOSD	neuromyelitis optica spectrum disorder
PFA	paraformaldehyde
PKB	protein kinase B
PMN	polymorphonuclear cell
RTCA	real-time cell analysis
SEM	standard error of the mean
TJ	tight junction
TNF	tumor necrosis factor
vWF	von Willebrand factor
ZO-1	zonula occludens-1
